# New Algorithms Improving PML Risk Stratification in MS Patients Treated With Natalizumab

**DOI:** 10.3389/fneur.2020.579438

**Published:** 2020-12-17

**Authors:** Inmaculada Toboso, Amalia Tejeda-Velarde, Roberto Alvarez-Lafuente, Rafael Arroyo, Harald Hegen, Florian Deisenhammer, Susana Sainz de la Maza, José C. Alvarez-Cermeño, Guillermo Izquierdo, Dolores Paramo, Pedro Oliva, Bonaventura Casanova, Eduardo Agüera-Morales, Diego Franciotta, Matteo Gastaldi, Oscar Fernández, Patricia Urbaneja, José M. Garcia-Dominguez, Fernando Romero, Alicia Laroni, Antonio Uccelli, Angel Perez-Sempere, Albert Saiz, Yolanda Blanco, Daniela Galimberti, Elio Scarpini, Carmen Espejo, Xavier Montalban, Ludwig Rasche, Friedemann Paul, Inés González, Elena Álvarez, Cristina Ramo, Ana B. Caminero, Yolanda Aladro, Carmen Calles, Pablo Eguía, Antonio Belenguer-Benavides, Lluis Ramió-Torrentà, Ester Quintana, José E. Martínez-Rodríguez, Agustín Oterino, Carlos López de Silanes, Luis I. Casanova, Lamberto Landete, Jette Frederiksen, Gabriel Bsteh, Patricia Mulero, Manuel Comabella, Miguel A. Hernández, Mercedes Espiño, José M. Prieto, Domingo Pérez, María Otano, Francisco Padilla, Juan A. García-Merino, Laura Navarro, Alfonso Muriel, Lucienne Costa Frossard, Luisa M. Villar

**Affiliations:** ^1^Immunology Department, Hospital Universitario Ramon y Cajal, Madrid, Spain; ^2^Instituto de Investigación Sanitaria San Carlos (IDISSC), Hospital Clinico San Carlos, Madrid, Spain; ^3^Department of Neurology, Hospital Universitario Quiron Salud, Madrid, Spain; ^4^Department of Neurology, Medical University of Innsbruck, Innsbruck, Austria; ^5^Neurology Department, Hospital Universitario Ramon y Cajal, Madrid, Spain; ^6^Neurology Department, Hospital Universitario Virgen Macarena, Sevilla, Spain; ^7^Neurology Department, Hospital Universitario Central de Asturias, Oviedo, Spain; ^8^Neurology Department, Hospital Universitario la Fe, Valencia, Spain; ^9^Neurology Department, Hospital Universitario Reina Sofia, Cordoba, Spain; ^10^Istituti di Recovero e Cura a Carattere Scientifico (IRCCS) Mondino Foundation, Pavia, Italy; ^11^Neurology Department, Hospital Regional Universitario, Malaga, Spain; ^12^Neurology Department, Hospital General Universitario Gregorio Marañón, Madrid, Spain; ^13^University of Genoa, Ospedale Policlinico San Martino, Genoa, Italy; ^14^Neurology Department, Hospital General Universitario de Alicante, Alicante, Spain; ^15^Neurology Service, Hospital Clinic and Institut d'Investigacions Biomèdiques August Pi i Sunyer (IDIBAPS), Universitat de Barcelona, Barcelona, Spain; ^16^Centro Dino Ferrari, Fondazione Ca' Granda, Istituti di Recovero e Cura a Carattere Scientifico (IRCCS) Ospedale Policlinico, University of Milan, Milan, Italy; ^17^Servei de Neurologia-Neuroimmunologia, Centre d'Esclerosi Múltiple de Catalunya, Vall d'Hebron Institut de Recerca, Hospital Universitari Vall d'Hebron, Universitat Autònoma de Barcelona, Barcelona, Spain; ^18^Department of Neurology, NeuroCure Clinical Research Center, Charité—Universitätsmedizin Berlin, Corporate Member of Freie Universität Berlin, Humboldt-Universität zu Berlin, Berlin Institute of Health, Berlin, Germany; ^19^Experimental and Clinical Research Center, Charité—Universitätsmedizin Berlin, Max Delbrück Center for Molecular Medicine, Berlin, Germany; ^20^Neurology Department, Hospital Alvaro Cunqueiro, Vigo, Spain; ^21^Neurology Department, Hospital Germans Trias i Pujol, Badalona, Spain; ^22^Neurology Department, Hospital Nuestra Señora de Sonsoles, Avila, Spain; ^23^Neurology Department, Hospital Universitario Getafe, Getafe, Spain; ^24^Neurology Department, Hospital Universitario Son Espases, Palma de Mallorca, Spain; ^25^Neurology Department, Hospital Doctor Jose Molina Orosa, Arrecife, Spain; ^26^Neurology Department, Hospital General Universitario de Castellón, Castellón, Spain; ^27^Neurology Department, Hospital Universitario Doctor Josep Trueta, Girona, Spain; ^28^Neurology Department, Hospital del Mar, Barcelona, Spain; ^29^Neurology Department, Hospital Universitario Marqués de Valdecilla, Santander, Spain; ^30^Neurology Department, Hospital Universitario de Torrejón, Torrejón de Ardoz, Spain; ^31^Neurology Department, Hospital Universitario Dr. Peset, Valencia, Spain; ^32^Glostrup Hospital, University of Copenhagen, Copenhagen, Denmark; ^33^Neurology Department, Hospital Universitario Nuestra Señora de Candelaria, Tenerife, Spain; ^34^Neurology Department, Hospital Clínico de Santiago, Santiago de Compostela, Spain; ^35^Neurology Department, Hospital del Bierzo, Ponferrada, Spain; ^36^Neurology Department, Complejo Hospitalario de Navarra, Pamplona, Spain; ^37^Neurology Department, Hospital Clinico de Malaga, Malaga, Spain; ^38^Neurology Department, Hospital Puerta de Hierro, Majadahonda, Madrid, Spain; ^39^Neurology Department, Hospital General de Elche, Elche, Spain; ^40^Biostatistics Unit, Hospital Univesitario Ramon y Cajal, Instituto Ramon y Cajal para la Investigación Sanitaria (IRYCIS), Madrid, Spain

**Keywords:** multiple sclerosis, demyelinating diseases, biomarkers, natalizumab, progressive multifocal leucoencephalopathy, disease modifying treatments

## Abstract

**Overview:** We assessed the role of age and disease activity as new factors contributing to establish the risk of progressive multifocal leucoencephalopathy in multiple sclerosis patients treated with natalizumab in 36 University Hospitals in Europe. We performed the study in 1,307 multiple sclerosis patients (70.8% anti-John Cunninghan virus positive antibodies) treated with natalizumab for a median time of 3.28 years. Epidemiological, clinical, and laboratory variables were collected. Lipid-specific IgM oligoclonal band status was available in 277 patients. Factors associated with progressive multifocal leucoencephalopathy onset were explored by uni- and multivariate logistic regression.

**Results:** Thirty-five patients developed progressive multifocal leucoencephalopathy. The multivariate analysis identified anti-John Cunninghan virus antibody indices and relapse rate as the best predictors for the onset of this serious opportunistic infection in the whole cohort. They allowed to stratify progressive multifocal leucoencephalopathy risk before natalizumab initiation in individual patients [area under the curve (AUC) = 0.85]. The risk ranged from <1/3,300 in patients with anti-John Cunninghan virus antibody indices <0.9 and relapse rate >0.5, to 1/50 in the opposite case. In patients with lipid-specific IgM oligoclonal bands assessment, age at natalizumab onset, anti-John Cunninghan virus antibody indices, and lipid-specific IgM oligoclonal band status predicted progressive multifocal leucoencephalopathy risk (AUC = 0.92). The absence of lipid-specific IgM oligoclonal bands was the best individual predictor (OR = 40.94). The individual risk ranged from <1/10,000 in patients younger than 45 years at natalizumab initiation, who showed anti John Cunningham virus antibody indices <0.9 and lipid-specific IgM oligoclonal bands to 1/33 in the opposite case.

**Conclusions:** In a perspective of personalized medicine, disease activity, anti-lipid specific IgM oligoclonal bands, anti Jonh Cunninghan virus antibody levels, and age can help tailor natalizumab therapy in multiple sclerosis patients, as predictors of progressive multifocal leucoencephalopathy.

## Introduction

The use of natalizumab, a highly effective therapy approved for the treatment of active relapsing-remitting multiple sclerosis (1), is limited by the risk of progressive multifocal leucoencephalopathy (PML), a serious opportunistic infection of the central nervous system caused by John Cunninghan virus (JCV), appearing in about 1/250 treated patients (2, 3).

The factors most frequently used to stratify PML risk in multiple sclerosis patients treated with natalizumab are the presence of anti-JCV antibodies or high anti-JCV indexes in serum; prior immunosuppressive therapies; and duration of natalizumab treatment (3–7). These factors have proven to be effective in reducing the risk of PML in the clinical setting (8, 9). However, these strategies present some limitations. They depend on treatment duration and anti-JCV antibody levels, or negative anti-JCV status may change a long time, and this modifies patient prognosis (10, 11). Therefore, the search for new factors to stratify PML risk is of great clinical relevance. A highly inflammatory disease, revealed by the presence of lipid-specific oligoclonal IgM bands (LS-OCMB) in cerebrospinal fluid (CSF), associates with a lower PML risk in natalizumab treated patients (10). However, it remains unknown if clinical data indicating high inflammatory course prior natalizumab onset can also predict PML risk.

It was also demonstrated that mean age is higher in multiple sclerosis patients suffering PML during natalizumab treatment (10, 12, 13). However, the role of age as PML risk factor has not been fully explored.

We studied in a multicenter cohort of multiple sclerosis patients treated with natalizumab whether patients' clinical and demographic characteristics can be useful in predicting PML onset. Moreover, we further investigated the utility of LS-OCMB for the stratification of PML risk in combination with other clinical and laboratory variables.

## Materials and Methods

This was a multicenter cross-sectional study including 1,307 patients treated with natalizumab (natalizumab treatment duration: 3.73 ± 2.13 years, mean ± *SD*) in 36 European hospitals. The study was approved by the ethical committee of Ramon y Cajal University Hospital. All patients signed and informed consent before entering.

Patients were followed every 3–6 months in the neurology clinics at every participating center, with additional visits in case of relapses. Demographic, clinical, and laboratory data prospectively collected at every center were anonymized and sent to the coordinator center. All patients signed an informed consent obtained according to the Declaration of Helsinki before entry.

### Inclusion Criteria

We established the following inclusion criteria:

Patients had to be treated with natalizumab for at least a year to avoid the effect of a short time of treatment as confounder factor.

Clinical data had to be obtained prospectively since disease onset to avoid the lack of accuracy of retrospective data acquisition.

### Data Collection

We established a minimum sample size of 1,000 patients to analyze all the variables projected. A form was sent to the participating centers comprising the following variables: sex, age at first relapse, age at natalizumab initiation, time between multiple sclerosis onset and natalizumab initiation, duration of natalizumab treatment, Expanded Disability Status Scale (EDSS) at natalizumab initiation, Multiple Sclerosis Severity Scale (MSSS) (14) at natalizumab initiation, relapse rate measured from multiple sclerosis onset to natalizumab initiation, previous treatments, serum anti-JCV antibody status (positive or negative), anti-JCV antibody index (which is proportional to serum anti-JCV antibody levels) (5), IgG oligoclonal bands (OCGB), and PML onset. LS-OCMB were available in a sub-cohort of 277 patients recruited at 29 different hospitals. LS-OCMB were determined by isoelectric focusing and immunoblotting, as previously described (15).

After receiving the first set of results, the database was debugged three times to complete data collection and correct inconsistent results. Finally, 69 patients were excluded, because of incomplete data or treatment duration shorter than 1 year. All the analyses were performed in the remaining 1,240 multiple sclerosis patients. Missing data were found in the following variables: Anti-John Cunninghan (JC) antibodies were only available in 1,174 patients (97.5%). Thirty-four of them developed PML, and 1,140 did not. Of note, in two PML cases anti-JC antibodies were negative 4 and 6 months before PML onset, when the last control test was performed. In both cases, the anti-JC test became positive at PML diagnosis. Anti-JC antibody levels were only available in 1,016 patients (82%). Twenty-seven developed PML, and 989 did not; relapse rate before natalizumab initiation was only obtained in 1,224 cases (98.7%). Thirty-five developed PML, and 1,189 did not. Finally, data on OCGB were only available in 756 patients (61%). Thirty-two developed PML, and 726 did not. Data collection comprised from 31 March 2017 to 15 June 2018.

### Statistical Analysis

Results were analyzed with STATA v.14 (StataCorp.2014. Statistical Software: Release 14. College Station, TX, USA). *p* < 0.05 were considered as significant.

Normality of the different variables in PML and not PML groups was assessed with Kolmogorov–Smirnov test. No variable passed normality test in PML group. Thus, Mann–Whitney *U*-test (two tailed) was applied for non-parametric tests and Fisher exact test (two sided) was used for comparisons of categorical variables between groups. Univariate tests based on logistic regression were used to explore variables associated to PML risk and to calculate odds ratios (OR) and confidence intervals (CI). Significant results obtained in the univariate analyses were explored by multivariate tests, and minimal models were established by eliminating variables loosing statistical significance.

To assess PML risk in individual patients, a nomogram was generated from the minimal model logistic regression results. In this analysis, the program assigns a score to every factor increasing PML risk. It also creates two parallel scales with total scores and the correspondent probability of PML. To explore individual risk in a patient, the total score is calculated and the corresponding risk read in the probability scale. To avoid overestimating PML risk, probabilities were corrected by a factor obtained by dividing previously described PML frequency in natalizumab treated patients (3) and the one obtained in our cohorts.

### Data Availability

The study protocol, statistical analysis plan, and data not provided in the article because of space limitations will be shared upon request by any qualified investigator for purposes of replicating procedures and results during 3 years after publication.

## Results

We included in the study 1,240 multiple sclerosis patients treated with natalizumab at 36 different hospitals. Thirty-five developed PML during natalizumab treatment, and 1,205 did not suffer this opportunistic infection. Clinical and demographic data of the patients classified according to PML onset are shown in Table 1A. The highest differences were found in age at natalizumab initiation (*p* = 0.004), relapse rate before natalizumab (*p* < 0.0001), anti-JCV antibody positivity (*p* = 0.004), and anti-JCV index levels (*p* < 0.0001). PML patients were older at treatment initiation, showed a lower relapse rate, a higher proportion tested positive for anti-JCV antibodies before PML, and had increased anti-JCV antibody indices. We also found that PML group showed an increased proportion of males (*p* = 0.04) and had longer disease duration at natalizumab initiation (*p* = 0.02). No variation in other clinical or demographic variables was associated with PML, including prior immunosuppression or duration of natalizumab treatment. We further explored if the values of this variable could change depending on anti-JC antibody values. The median time of treatment was 3.28 years in the whole cohort, the range going from 1.00 to 13.40 years, and the interquartile range (IQR) from 2.06 to 4.82 years. These values did not change substantially in patients with anti-JC antibody levels higher (median = 3.32, range: 1.00–11.46, IQR: 2.01–5.03 years) or lower (median = 3.26, range: 1.00–13.40, IQR: 2.08–4.3 years) than 0.9.

**Table 1:** Demographic and clinical data.

	**(A) Total group (*N* = 1,240)**			**(B) LS-OCMB group (*N* = 277)**		
	**Pml (*n* = 35)**	**NoT PML (*n* = 1,205)**	***P***	**PML (*N* = 24)**	**NOT PML (*N* = 253)**	***p***
Sex (M/F)	16/19	358/847	0.04	10/14	78/175	0.28
Age at 1st relapse (y)	30.1 ± 9.5 (23–36)	28.2 ± 8.7 (22–33)	0.33	31.1 ± 9.6	28.1 ± 8.41	0.14
Disease duration at NTZ onset (y)	11.2 ± 7.4 (4.7–17.9)	8.3 ± 6.3 (3.4–11.9)	0.02	12.7 ± 7.8	6.7 ± 5.7	0.0002
Age at NTZ onset (y)	41.3 ± 8.9 (33.2–49.2)	36.5 ± 9.4 (29.9–42.7)	0.004	43.8 ± 8.7	34.8 ± 8.9	<0.0001
Duration of NTZ treatment (y)	3.4 ± 1.5 (1.1–7.7)	3.8 ± 2.1 (1.0–13.4)	0.77	3.3 ± 1.6	3.47 ± 2.0	0.82
EDSS at NTZ onset	3.3 ± 1.4 (2–4)	3.2 ± 1.6 (2–4)	0.68	3.6 ± 1.4	3.1 ± 1.6	0.07
MSSS at NTZ onset	4.3 ± 2.5 (2.2–6.8)	4.8 ± 2.4 (2.8–6.6)	0.24	4.4 ± 2.6	5.1 ± 2.4	0.23
Relapse rate before NTZ onset	0.8 ± 0.95 (0.25–0.93)	1.4 ± 1.4 (0.53–1.56)	<0.0001	0.6 ± 0.5	1.6 ± 1.7	<0.0001
Prior IS (yes/no)	7/28	139/1066	0.13	5/19	34/219	0.32
Anti-JCV Abs (pos/neg)^*^	32/2	844/331	0.004	21/2	162/87	0.010
Anti-JCV Ab levels^*^	2.2 ± 1.2 (1.23–3.18)	0.9 ± 1.1 (0.09–1.45)	<0.0001	1.9 ± 1.3	1.0 ± 1.1	0.0047
OCGB (pos/neg)	30/2	651/73	0.48	22/2	234/19	0.88
LS-OCMB (pos/neg)				1/23	162/91	<0.0001

*For continuous variables values are expressed as mean ± standard deviation (interquartile range)*.

* *The last measure before study completion; 1^st^, first; Anti-JCV Ab, anti-John Cunningham virus antibodies; EDSS, expanded disability status scale; F, female; IS, immunosuppression; LS-OCMB, lipid-specific oligoclonal IgM bands; M, male; MSSS, multiple sclerosis severity score; neg, negative; NOT PML, not progressive multifocal leukoencephalopathy; NTZ, Natalizumab; OCGB, oligoclonal IgG bands; PML, progressive multifocal leukoencephalopathy; pos, positive; y, years*.

To better define associations of the different variables with PML onset, we first performed univariate analyses (Table 2A). Cutoff values were established using receiver operating characteristic (ROC) curves in case of age, time until natalizumab initiation, and relapse rate before treatment or pre-established cutoffs for anti-JCV antibody levels and EDSS and MSSS scores. The strongest association was found with high anti-JCV index values, being the clearest one obtained for anti-JCV indices higher than 0.9 (OR = 18.29, *p* < 0.001). Additionally, having anti-JCV index levels >1.5 (OR = 8.58, *p* < 0.001) and the presence of anti-JCV antibodies (OR = 6.27, *p* = 0.012) also associated with PML onset. Age at natalizumab initiation ≥45 years also increased PML risk (OR = 3.20, *p* = 0.001). A disease duration higher than 10 years at natalizumab initiation (OR = 2.37, *p* = 0.012) and an MSSS score lower than 3 (OR = 2.25, *p* = 0.019) was also associated with PML onset. Finally, male sex associated modestly with an increased PML risk (OR = 1.99, *p* = 0.046). On the other hand, having an annualized relapse rate higher than 0.5 before treatment initiation clearly diminished PML risk (OR = 4.47, *p* < 0.001).

**Table 2:** Univariate analysis to explore the ability of different clinical and demographic variables for predicting PML onset during natalizumab treatment.

	**(A) Total patient group (*****n*** **= 1,240)**			**(B) Patients with a LS-OCMB study (*****n*** **= 277)**		
	**OR**	**95% CI**	***P***	**OR**	**95% CI**	***p***
Male sex	1.99	1.06–4.17	0.046	1.60	0.68–3.77	0.28
Age at NTZ onset ≥45 years	3.20	1.60–6.39	0.001	7.36	3.06–17.72	<0.001
Disease duration at NTZ onset ≥10 years	2.37	1.21–4.66	0.012	4.60	1.94–10.90	0.001
NTZ treatment for >2 years	1.02	0.46–2.27	0.96	1.69	0.61–4.70	0.31
NTZ treatment for >3 years	1.25	0.63–2.48	0.53	1.29	0.56–2.99	0.55
NTZ treatment for >4 years	0.99	0.43–1.98	0.97	0.91	0.36–2.27	0.84
NTZ treatment for >5 years	0.43	0.15–1.23	0.12	0.41	0.09–1.80	0.24
Positive anti–JCV Abs	6.27	1.50–26.33	0.012	5.64	1.29–24.61	0.021
Anti-JCV Ab levels ≥0.9	18.29	5.46–61.19	<0.001	9.08	2.54–32.45	0.001
Anti-JC Ab levels ≥1.5	8.58	3.59–20.54	<0.001	4.78	1.71–13.33	0.003
EDSS at NTZ onset <3	0.75	0.38–1.51	0.42	0.43	0.17–1.07	0.07
EDSS at NTZ onset <6	1.15	0.35–3.82	0.82	1.31	0.29–5.90	0.72
MSSS at NTZ onset <3	2.25	1.14–4.43	0.019	2.25	0.95–5.32	0.06
MSSS at NTZ onset <6	0.95	0.47–1.94	0.90	0.86	0.35–2.09	0.74
Relapse rate before NTZ onset <0.5	4.47	2.26–8.86	<0.001	6.77	2.80–16.35	<0.001
Prior immunosuppression	1.92	0.82–4.47	0.13	1.70	0.59–4.84	0.32
LS-OCMB Negative				40.94	5.44–308.20	<0.001

*Anti-JCV Abs, anti-John Cunningham virus antibodies; CI, confidence interval; EDSS, expanded disability status scale; LS-OCMB, lipid-specific oligoclonal IgM bands; MSSS, multiple sclerosis severity score; NTZ, natalizumab; OR, odd ratio; PML, progressive multifocal leukoencephalopathy*.

Based on univariate analyses, we performed three different multivariate analyses according to anti-JCV antibody classification. First, we included all the significant factors and anti-JCV antibodies classified according to the positive or negative results (Table 3). In the minimal model, anti-JCV antibody positivity (OR = 6.04, *p* = 0.014), annualized relapse rate before natalizumab <0.5 (OR = 4.25, *p* < 0.001), and age at natalizumab initiation ≥45 years (OR = 2.33, *p* = 0.022) significantly impacted on PML appearance. In this model area under the ROC curve was 0.78.

**Table 3:** Factors predicting PML onset in the total group of patients.

	**OR**	**95% CI**	***P***
**Minimal model with anti-JCV antibodies classified as positive/negative**			
LS-OCMB negative Age at natalizumab initiation ≥45 years Relapse rate before natalizumab initiation <0.5	30.44 4.80 3.21	3.94–234.91 1.76–13.14 1.19–8.66	<0.001 0.002 0.022
Area under ROC curve: 0.90			
**Minimal model with anti-JCV antibodies classified using a level of 0.9 as cut off value**			
LS-OCMB negative Age at natalizumab initiation ≥45 years Anti-JCV antibodies levels ≥0.9	26.83 6.74 6.52	3.33–216.29 2.00–22.73 1.64–25.85	0.002 0.002 0.008
Area under ROC curve: 0.92			
**Minimal model with anti-JCV antibodies classified using a level of 1.5 as cut off value**			
LS-OCMB negative Age at natalizumab initiation ≥45 years Anti-JCV antibodies levels ≥1.5	31.18 8.85 4.38	3.81–255.16 2.64–29.60 1.33–14.43	0.001 <0.001 0.015
Area under ROC curve: 0.92			

*Multivariate analyses*.

*Anti-JCV antibodies, anti-John Cunningham virus antibodies; CI, confident interval; LS-OCMB, lipid-specific oligoclonal IgM bands; OR, odd ratio; PML, progressive multifocal leukoencephalopathy; ROC, Receiver operating characteristic*.

The second multivariate analysis included anti-JCV antibodies classified using the level of 0.9 as cutoff value. In the minimal model, only anti-JCV antibody levels ≥0.9 (OR = 18.72, *p* < 0.001) and annualized relapse rate before natalizumab initiation <0.5 (OR = 4.66, *p* < 0.001) had an effect on PML risk. Although only these two factors were significant in this model, the area under ROC curve was higher (0.85).

Finally, we performed a multivariate analysis using 1.5 as cutoff value for anti-JC antibody levels. Anti-JCV antibody levels ≥1.5 (OR = 7.85, *p* < 0.001), annualized relapse rate before natalizumab initiation <0.5 (OR = 3.73, *p* = 0.001), and age at natalizumab initiation ≥45 years (OR = 2.31, *p* = 0.048) significantly increased PML risk in this model. The area under the ROC curve was 0.84.

**Figure 1:**
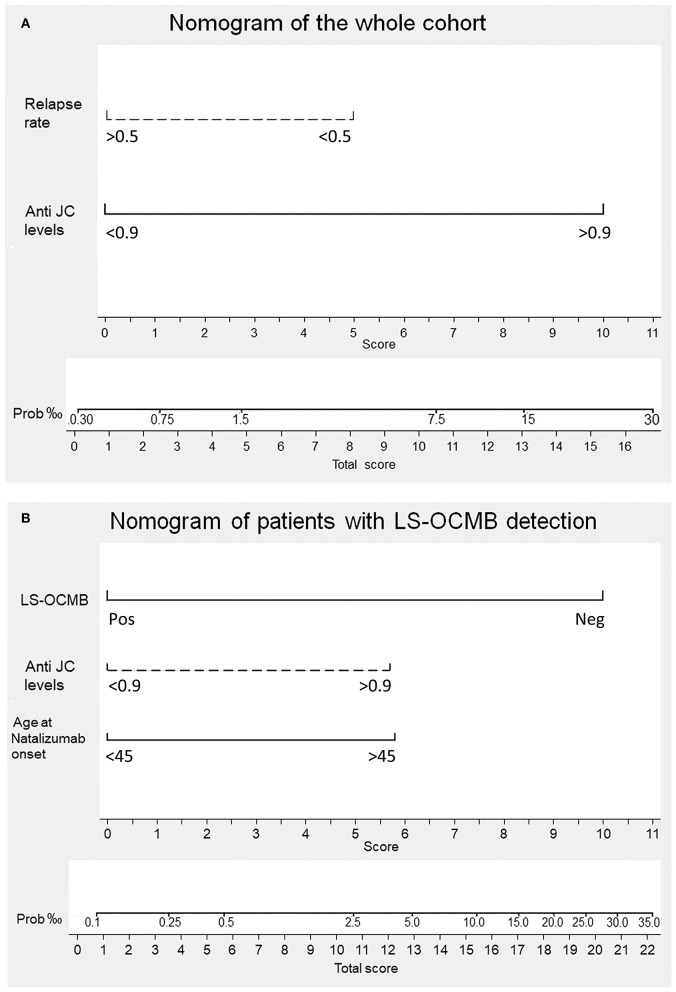
Nomogram for predicting progressive multifocal leukoencephalopathy (PML) onset in individual MS patients. The multivariate logistic regression analysis assigns a score to every variable included in the minimal model. The sum of the scores obtained by a patient is interpolated in the total score point-probability line at the bottom of each nomogram and gives the individual PML risk. **(A)** PML risk in the total cohort. Having a relapse rate lower than 0.5 gives a score of 5 and showing anti-John Cunningham virus antibody levels (anti-JC levels) higher than 0.9 provides a score of 10. Individual patient scores range from 0 to 15 and their PML risk from <1/3,300 to 1/50, respectively. **(B)** PML risk in the patients with lipid-specific oligoclonal IgM band (LS-OCMB) detection. Being negative (Neg) for LS-OCMB gives a score of 10. Showing anti-JC levels higher than 0.9 provides a score of 5.75. Being older than 45 years gives a score of 5.75. Individual patient scores range from 0 to 21.5 and their PML risk from <1/10,000 to 1/30, respectively.

We made a nomogram analysis of the second multivariate analysis (cutoff: anti-JCV antibody levels of 0.9) to explore the contribution of each variable to PML risk. Data are shown in Figure 1A. We adjusted the risk using a correction factor obtained calculating the ratio between the numbers of PML cases per 1,000 patients reported after commercialization (4.16‰) and that of our cohort (28‰). Patients with anti-JCV antibody levels lower than 0.9 and annualized relapse rate higher than 0.5 prior natalizumab initiation showed a PML risk lower than 0.3‰. If the annualized relapse rate was lower than 0.5, the PML risk increased to 1.5‰, and if, in addition, anti-JCV antibody levels were higher than 0.9, the risk was augmented to 2%. These values are independent of the sex, disease duration, time on natalizumab treatment, or previous treatment with anti-suppressive drugs.

### Role of Lipid Specific Oligoclonal IgM Bands in Risk Stratification

Two hundred seventy-seven patients (22.3% of the whole cohort) were examined for LS-OCMB. Twenty-four of them (8.7%) developed PML. Clinical and demographic data of these patients are shown in Table 1B. One hundred and sixty-two of the 253 patients not developing PML (64.0%) were LS-OCMB positive. By contrast, only one of the 24 PML patients (4.2%) displayed these antibodies (*p* < 0.0001). Similarly to the whole cohort, patients suffering PML were older (*p* < 0.0001), had a longer disease duration (*p* = 0.0002) at natalizumab initiation, and had a lower relapse rate before natalizumab (*p* < 0.0001). A higher percentage of these patients were anti-JCV positive (*p* = 0.010), and they also displayed higher anti-JCV antibody levels (*p* = 0.0047).

We followed the same approach described for the entire cohort. First, we performed univariate analyses (Table 2B). The conditions associated with PML risk were the following: absence of LS-OCMB (OR = 40.94; *p* < 0.001); levels of anti-JCV index ≥0.9 (OR = 9.08, *p* = 0.001) or ≥1.5 (OR = 4.78, *p* = 0.003); or positive anti-JCV antibodies (OR = 5.64, *p* = 0.021); age at natalizumab initiation ≥45 years (OR = 7.36, *p* < 0.001); annualized relapse rate before natalizumab ≤0.5 (OR = 6.77, *p* < 0.001), and disease duration at natalizumab initiation ≥10 years (OR = 4.60, *p* = 0.001).

Again, we made three different multivariate models according to anti-JCV antibody classification (Table 4). First, we included all the significant variables and anti-JCV antibodies classified according to having positive or negative results. In the minimal model, only absence of LS-OCMB (OR = 30.44, *p* < 0.001), age at natalizumab initiation ≥45 years (OR = 4.80, *p* = 0.002), and relapse rate before natalizumab initiation <0.5 (OR = 3.21, *p* = 0.022) had an effect on PML risk. The area under the ROC curve was 0.90.

**Table 4:** Factors predicting PML onset in the group of patients with LS-OCMB detection.

	**OR**	**95% CI**	***P***
**Minimal model with anti-JCV antibodies classified as positive/negative**			
Anti-JCV antibodies (positive) Relapse rate before natalizumab onset <0.5 Age at natalizumab onset ≥45 years	6.04 4.25 2.33	1.43–25.53 2.08–8.69 1.13–4.80	0.014 <0.001 0.022
*Area under ROC curve: 0.78*			
**Minimal model with anti-JCV antibodies classified using a level of 0.9 as cut off value**			
Anti-JCV antibody levels ≥0.9 Relapse rate before natalizumab onset <0.5	18.7 4.66	5.56–63.02 2.10–10.35	<0.001 <0.001
*Area under ROC curve: 0.85*			
**Minimal model with anti-JCV antibodies classified using a level of 1.5 as cut off value**			
Anti-JCV antibodies levels ≥1.5 Relapse rate before natalizumab onset <0.5 Age at natalizumab onset ≥45 years	7.85 3.73 2.31	3.25–19.00 1.67–8.34 1.01–5.28	<0.001 0.001 0.048
*Area under ROC curve: 0.84*			

*Multivariate analyses*.

*Anti-JC antibodies, anti-John Cunningham virus antibodies; CI, confident interval; OR, odd ratio; PML, progressive multifocal leukoencephalopathy; ROC, Receiver operating characteristic*.

In the second multivariate analysis we used an anti-JCV index of 0.9 as cutoff value. In the minimal model the variables that significantly impacted PML development were absence of LS-OCMB (OR = 26.83, *p* = 0.002), age at natalizumab initiation ≥45 years (OR = 6.74, *p* = 0.002), and anti-JCV antibody index ≥0.9 (OR = 6.52, *p* = 0.008). In this model, the area under the ROC curve was 0.92.

Finally, we performed a multivariate analysis using 1.5 as cutoff value for anti-JCV antibody levels. Again, absence of LS-OCMB (OR = 31.18, *p* = 0.001), age at natalizumab initiation ≥45 years (OR = 8.85, *p* < 0.001), and anti-JCV antibody levels ≥1.5 (OR = 4.38, *p* = 0.015) significantly increased PML risk. The area under the ROC curve was 0.92.

Finally, we repeated a nomogram analysis of the second multivariate analysis (cutoff: anti-JCV antibody levels of 0.9). Data are shown in Figure 1B. We adjusted the risk using a correction factor obtained calculating the ratio between the numbers of PML cases per 1,000 patients reported after commercialization (4.16‰) and that of our cohort (87‰). Patients with LS-OCMB, anti-JCV antibody levels lower than 0.9, and age at natalizumab initiation younger than 45 years showed a PML risk lower than 0.1‰. When anti-JCV antibody levels were higher than 0.9 or patients were older than 45 at natalizumab onset, the risk was augmented to 0.5‰. If both conditions were present, it rose to 3.5‰. If LS-OCMB were negative too, the risk increased to 3%. Again, these values were independent of sex, disease duration, prior immunosuppression, or the duration of natalizumab treatment.

**Figure 2:**
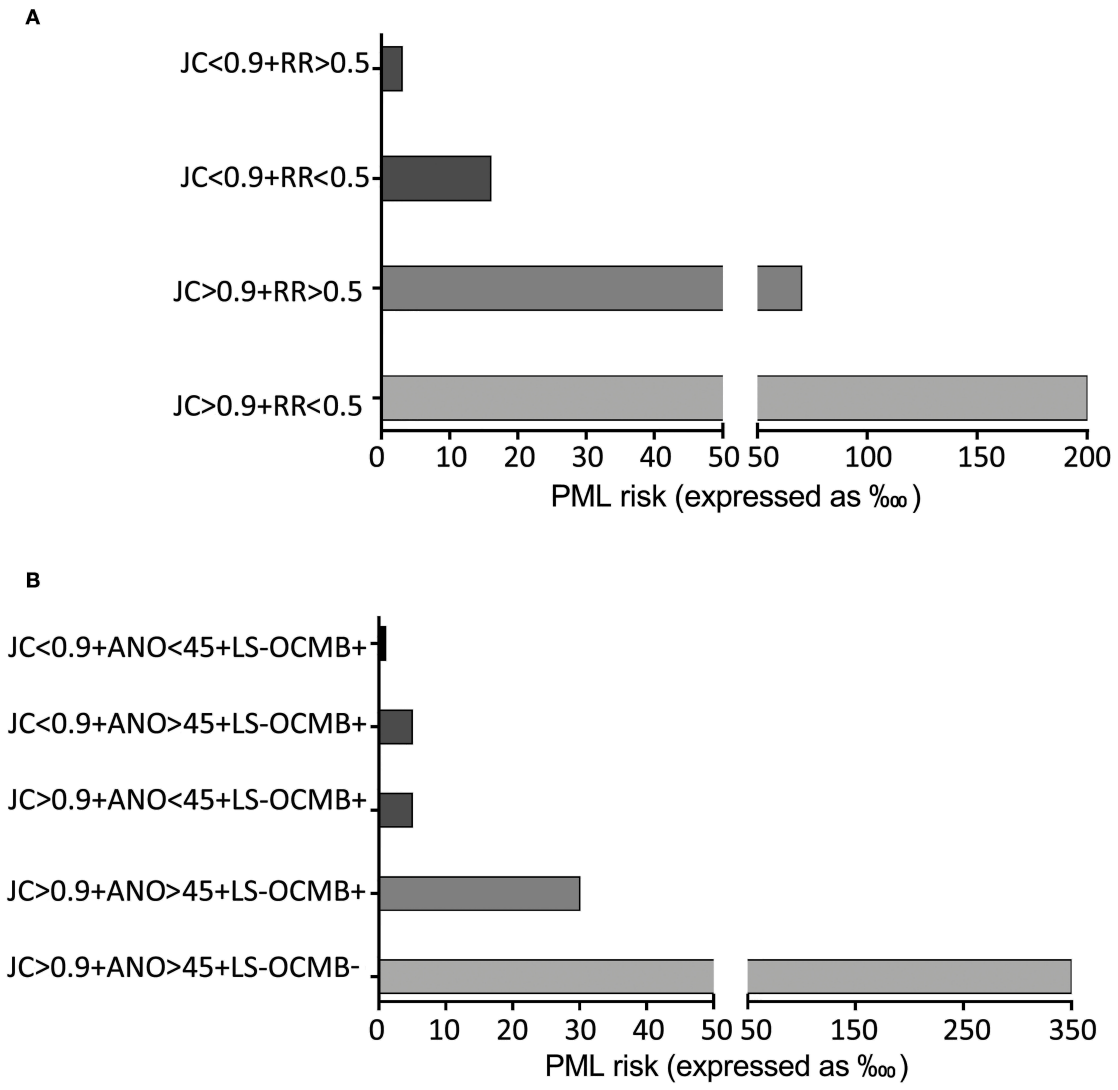
Illustration of predicting progressive multifocal leukoencephalopathy (PML) risk depending on the results of the nomograms. **(A)** In the whole cohort PML risk associates with the anti-John Cunningham virus antibody levels (JC) and the relapse rate (RR). **(B)** In patients with lipid-specific oligoclonal IgM band (LS-OCMB) detection, PML risk associated with the LS-OCMB, and JC status, and the age at natalizumab onset (ANO).

A graphic representation of PML risk in the two cohorts depending of the results of the nomograms is shown in Figure 2.

Finally, we studied if OCMB could add some advantage to previous risk factors in the 47 patients (29 female/19 male) with anti-JCV antibody levels >1.5, who were treated with natalizumab for more than 2 years (2.32, 2.01–4.32 years; median, interquartile range). Results are shown in Table 5. Only one of 23 patients showing OCMB developed a PML. By contrast, 10 out of 24 OCMB negative patients suffered this opportunistic infection (Pearson chi square = 9.12, *p* = 0.003).

**Table 5:** Value of OCMB for predicting PML onset in patients with anti JC antibody levels >1.5 and treated with natalizumab for more than 2 years.

	**PML+**	**PML-**
LS-OCMB+ (*n*, %)	1, 4.35%	22, 95.65%
LS-OCMB- (*n*, %)	10, 41.67%	14, 58.33%
Total (*n*, %)	11, 23.40%	36, 76.60%
Pearson chi^2^ = 9.12 *p* = 0.003		

*LS-OCMB, lipid-specific oligoclonal IgM bands; PML, progressive multifocal leukoencephalopathy*.

## Discussion

The appearance of highly effective immunotherapies has changed disease course of patients with aggressive multiple sclerosis (1, 16–18). However, efficacy associates with higher risk of deleterious side effects (19–22). Finding biomarkers that allow the best balance between efficacy and safety for individual patients has become a challenge of the most clinical relevance in multiple sclerosis research.

In case of natalizumab, the most important side effect is the appearance of PML, an opportunistic infection of the brain appearing in about one of every 250 treated patients (3, 23). It may cause patient death or considerable increase of disability. This has limited the use of this drug. Safety concerns, in both patient and neurologist sides, often make it difficult to administer this treatment for long. This is unfortunate, since the clinical efficacy of this drug in the long term was demonstrated (24).

Additional factors reflecting patient inflammatory status can contribute to further stratify PML risk. Decreased CD4+ T cell expression of L-Selectin (CD62L), a molecule implicated in leukocyte adhesion to the endothelium, during natalizumab treatment was found to associate with an increase of PML risk (25). Although validation studies gave no uniform results (26, 27), probably due to the difficulty of measuring this biomarker in cryopreserved cells, these data may reflect that a decrease in cells migrating to the central nervous system may increase PML risk. Another factor indicating that patient inflammatory status may contribute to stratifying PML risk is one of the actual risk factors, prior immunosuppression. Previous treatments inducing a strong immunosuppression increase PML risk (4, 6).

Age, another factor associated with inhibition of the adaptive immune response in multiple sclerosis and with reduced lymphocyte migration into the central nervous system (CNS), also relates to a higher PML risk in multiple sclerosis patients treated with different biological drugs (10–12, 28). By contrast, a highly inflammatory disease course revealed by the presence of LS-OCMB greatly diminishes PML risk (10). We studied here if clinical data reflecting disease activity may contribute to stratify PML risk in a cohort of 1,240 patients treated with natalizumab in 36 European hospitals. We also studied the value of these variables in combination with LS-OCMB in a sub-cohort of 277 patients in which these antibodies were analyzed.

We did not find any significant association between prior immunosuppression and PML risk in our cohort, although the proportion of patients showing prior immunosuppression was higher in the group of PML patients (20%) than in those not developing this opportunistic infection (11%). The lower number of immunosuppressed patients in both PML and not PML cases compared with previous studies (5) may account for the lack of significance of this variable in our cohort. However, anti-JCV antibodies and mostly anti-JCV indices higher than 0.9 continued to increase the probability of PML in these patients. In addition, clinical data associated with disease activity also contribute to identify patients at higher risk. Thus, an MSSS score lower than 3 or relapse rates lower than 0.5 since disease onset is associated with increased probability of PML. Age older than 45 years at natalizumab onset also identified patients at higher PML risk. When including all factors giving significant results in the total cohort, in a multivariate logistic analysis to identify variables that were statistically independent, the best predictive model to assess PML risk included anti-JCV levels higher than 0.9 and annualized relapse rate below 0.5. We assessed individual PML risk by a nomogram analysis. When anti-JCV levels where below 0.9 and relapse rate over 0.5, PML risk was below 1 in every 3,300 treated patients. If the results were the opposite, it rose to 1/50.

By contrast, natalizumab treatment duration did not associate with PML risk in our study. The divergence of these results with those previously published can be partly due to the absence of patients treated for less than a year, who have extremely low PML risk, in our cohort. The relatively low number of patients included in this study (1,306) compared to other cohorts with more than 5,000 patients (5) may also contribute to the loss of significance of treatment duration for PML risk stratification. In addition, the particular characteristics of our cohort which includes mainly active (median relapse rate 0.88 with a low interquartile range of 0.51) and relatively young patients (median age at natalizumab onset = 36.5 years, with a high interquartile range of 42.8 years) also can contribute to the loss of significance in this variable. If these data are confirmed in larger cohorts, they could indicate that treatment duration impact on PML risk could be modulated by younger age and high disease activity.

The presence of LS-OCMB further contributed to stratify PML risk. When we performed a multivariate logistic analysis in the sub-cohort of patients in which these antibodies were assessed, the best predictive model to assess PML risk changed. It included LS-OCMB as best individual predictor and anti-JCV levels higher than 0.9 and age older than 45 years as factors that equally contributed to PML risk. Nomogram analysis showed that patients with CSF restricted LS-OCMB, anti-JCV antibodies below 0.9, and age younger than 45 years at natalizumab onset had a PML risk below 1 in every 10,000 treated patients. If anti-JCV antibody levels were higher than 0.9 or age at natalizumab onset over 45 years, PML risk was only 1/2,000 in LS-OCMB positive patients. When these two factors coincided in a patient, the risk rose to 1/300 despite LS-OCMB positivity and even increased to 1/33 in LS-OCMB negative patients. These data are clinically relevant since they show that patients with a more inflammatory disease, who get more clinical benefit of this highly active drug, are at lower PML risk during natalizumab treatment.

In conclusion, these data allow to introduce a new algorithm in which PML risk can be established for individual patients attending to clinical and laboratory data measured prior to natalizumab treatment initiation.

## Data Availability Statement

The raw data supporting the conclusions of this article will be made available by the authors, without undue reservation.

## Ethics Statement

The studies involving human participants were reviewed and approved by Ethics Committee of Hospital Ramon y Cajal, Madrid, Spain. The patients/participants provided their written informed consent to participate in this study.

## Author Contributions

LV, IT, RA-L, RA, HH, FD, SS, JA-C, GI, DPa, PO, BC, EA-M, DF, MG, OF, PU, JG-D, FR, AL, AU, AP-S, AS, YB, DG, ES, CE, XM, LR, FPau, IG, YA, EÁ, CR, AC, CC, PE, AB-B, LR-T, EQ, JM-R, AO, CL, LC, LL, JF, GB, PM, MH, JP, DPé, MO, FPad, JG-M, LN, AM, LF, and MC: sample collection, collection of clinical data, and critical review of the manuscript. All authors contributed to the article and approved the submitted version.

## Conflict of Interest

LV received a research grant from Biogen. The remaining authors declare that the research was conducted in the absence of any commercial or financial relationships that could be construed as a potential conflict of interest.
